# *Ignavigranum ruoffiae*, a rare pathogen that caused a skin abscess

**DOI:** 10.1099/jmmcr.0.005137

**Published:** 2018-01-16

**Authors:** Adriana N. De Paulis, Eugenia Bertona, Miguel A. Gutiérrez, María S. Ramírez, Carlos A. Vay, Silvia C. Predari

**Affiliations:** ^1^​ Departamento de Microbiología, Instituto de Investigaciones Médicas Alfredo Lanari, Universidad de Buenos Aires, Ciudad Autónoma de Buenos Aires, Argentina; ^2^​ Department of Biological Science, California State University Fullerton, Fullerton, CA, USA; ^3^​ Laboratorio de Bacteriología, Departamento de Bioquímica Clínica, Hospital de Clínicas José de San Martín, Facultad de Farmacia y Bioquímica, Universidad de Buenos Aires, Ciudad Autónoma de Buenos Aires, Argentina

**Keywords:** *I. ruoffiae*, skin abscess, rare pathogen

## Abstract

**Introduction:**

*Ignavigranum ruoffiae* is an extremely rare cause of human infections.

**Case presentation:**

An 83-year-old male with a painless, ten-day-old, erythematous skin abscess on his left flank, which had showed a purulent discharge for 48 h, was admitted to the Emergency service. He was treated with cephalexin, disinfection with Codex water and spray of rifampicin. Five days later, surgical drainage of the abscess was proposed due to the torpid evolution of the patient. Samples were taken for culture, and antibiotic treatment with trimethoprim-sulfamethoxazole was established. The patient returned after 10 days showing healing of the abscess. Microbiological studies showed a few Gram-positive cocci present as single cells and short chains that grew after 72 h of incubation at 35 °C with CO_2_ on 5 % sheep blood agar. Colonies presented a strong sauerkraut odour. Initial biochemical test results were negative for catalase, aesculin and bile-aesculin, and positive for pyrrolidonyl arylamidase, leucine aminopeptidase and growth in 6.5 % NaCl broth, which prompted the preliminary identification of *Facklamia* species or *I. ruoffiae*. The positive result for arginine deamination and negative result for hippurate hydrolysis, failure to produce acid from mannitol, sucrose, sorbitol or trehalose, plus the distinctive sauerkraut odour identified the organism as *I. ruoffiae*. The phenotypic identification was confirmed by 16S rRNA gene sequence analysis. The strain seemed to be susceptible to the antimicrobials tested but had decreased susceptibility to carbapenems.

**Conclusion:**

This case provides more insights into the phenotypic characteristics and antimicrobial resistance profile of *I. ruoffiae*.

## Introduction

The infections caused by *Ignavigranum ruoffiae* are extremely rare. The habitat of *I. ruoffiae* is not known; however, it can be inferred that *I. ruoffiae* is part of the resident microbiota of the skin. Few cases reported in the literature have been related to human skin infections. *I. ruoffiae* can be considered an opportunistic human pathogen and was first described by Collins *et al.* [1]. These bacteria should be characterized when they are isolated from normally sterile body sites and fluids, or in pure culture or as the predominating organisms from abscesses, or when they are isolated more than once from the same infection site [2].

Only three strains of *I. ruoffiae* obtained from the culture collection of the *Streptococcus* Laboratory at the Centers for Disease Control and Prevention, USA, have been described [1–4]. Strain 1607–97 was recovered from a wound infection and strain 3955–95 was isolated from an ear abscess. Both strains have been deposited in the culture collection of the University of Göteborg (CCUG), Sweden, under accession numbers CCUG 37658^T^ and CCUG 37841, respectively. The third strain was from an unspecific human source.

Our case report here is aimed to provide additional information regarding the phenotypic characteristics and the antimicrobial susceptibility profile of this rare micro-organism.

## Case presentation

An 83-year-old male with a painless, ten-day-old, erythematous skin abscess on his left flank, which had showed a purulent discharge for 48 h, was admitted to the Emergency service. He did not present fever or chills; however, a history of hypertension, coronary heart disease, dyslipidemia since 1996, being a former smoker and chronic renal failure were reported. The clinical examination showed an erythematous pustular lesion with purulent secretion and painless underlying induration. He was evaluated together with the Dermatology service and was treated with cephalexin 2 g per day for 7 days, disinfection with Codex water and spray of rifampicin. The patient was instructed to return to the Emergency service if he presented pain, redness, inflammation, haemorrhage, signs of infection including fever and chills, nausea and vomiting, dizziness and light-headedness, fast and irregular heartbeats, chest pain, rash or urticaria, because he was an anticoagulated patient. Five days later, the abscess was still draining pus and the Dermatology service evaluated the possibility of surgical drainage. On examination, the patient was afebrile with normal pressure, haemodynamically stable, and he presented a mass of 2×3 cm, draining purulent material. Surgical drainage of the abscess was proposed due to the torpid evolution of the patient despite being treated with cephalexin and rifampicin. Samples were taken for culture and pathology, and antibiotic treatment with trimethoprim-sulfamethoxazole (TMS) 800/160 mg for 5 days was established. The patient returned after 10 days showing healing of the abscess. The pathology biopsy results demonstrated an abscessed epidermal inclusion cyst with Gram-positive cocci arranged in short chains.

**Fig. 1.**
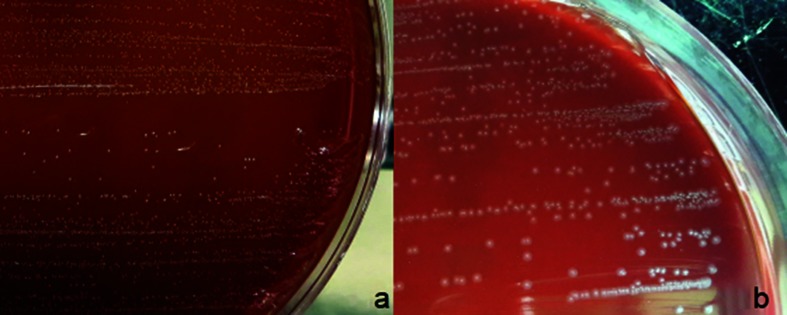
(a) *I. ruoffiae* on blood agar. Colonies were transparent and γ-haemolytic, of 1–2 mm in diameter after 72 h of incubation at 35 °C with CO_2_ on 5 % sheep blood agar. (b) After 5 to 7 days of incubation, *I. ruoffiae* colonies reached approximately 3 mm in diameter, with irregular borders and a slightly raised and denser centre.

**Fig. 2.**
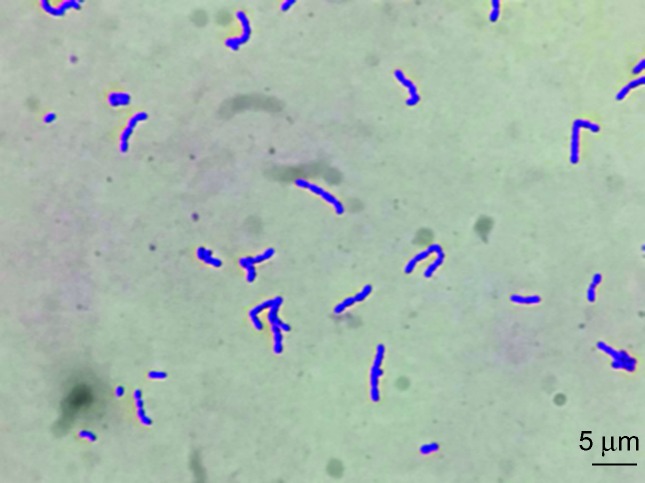
Gram staining of *I. ruoffiae* in fluid thioglycollate medium showed Gram-positive cocci present as single cells, pairs and short chains. The bar indicates 5 µm.

The Gram stain of the primary specimen showed a few Gram-positive cocci present as single cells and short chains, and Giemsa stain demonstrated 5–10 polymorphonuclear neutrophils per high-power field. The specimen was inoculated on 5 % sheep blood agar (Laboratorios Britania, Buenos Aires, Argentina) and chocolate agar incubated at 35 °C with the addition of 5 % CO_2_, and growth of a pure bacterial culture was observed after 48–72 h. Colonies were transparent and γ-haemolytic, of 1 to 2 mm in diameter, and reeked with a strong sauerkraut odour (Fig. 1a). With prolonged incubation (up to 7 days), colonies reached ≅ 3 mm in diameter, with irregular borders and a slightly raised and denser centre (Fig. 1b). The biochemical tests used in our laboratory for the phenotypic identification of the isolate were performed using conventional methods [3, 4]. The Gram staining of the sample and of the fluid thioglycollate medium showed cocci arranged as single cells, in pairs and as short chains (Fig. 2).

Preliminary test results showed that the isolate was vancomycin-sensitive, negative for catalase, gas from MRS glucose broth, aesculin and bile-aesculin, and positive for pyrrolidonyl arylamidase (PYR), leucine aminopeptidase (LAP) and growth with 6.5 % NaCl. In addition, the organism tested positive for arginine deamination, but failed to hydrolyse hippurate or produce acid form sucrose, sorbitol, mannitol or trehalose (Table 1).

**Table 1.** Phenotypic characteristics of *I. ruoffiae* and *Facklamia* species as shown by conventional biochemical tests The results obtained with the strain described in this paper are indicated in brackets. v, Feature variable; +^w^ result usually positive, sometimes weakly positive; nd: no data available. This table was adapted from the reports by Facklam [3] and LaClaire *et al.* [4].

Species	Hydrolysis of:		Acid production from:				
	Arginine*	Hippurate	Mannitol	Sucrose	Sorbitol	Trehalose	
*I. ruoffiae*	v[+]	− [−]	− [−]	− [−]	− [−]	− [−]	
*F. hominis*	+	+	−	v	−	−	
*F. ignava*	−	+	−	−	−	+^w^	
*F. languida*	−	−	−	−	−	+	
*F. sourekii*	−	+	−	+	+	v	
*F. tabacinasalis*	−	−	−	+	+	nd	

*Moeller decarboxylase basal medium with 1 % arginine was used.

Mass spectra were acquired using a matrix-assisted laser desorption/ionization time-of-flight mass spectrometry (MALDI-TOF MS) spectrometer in a linear positive mode (Microflex, Bruker Daltonics). The bacterial test standard (BTS, Bruker) was used for instrument calibration. Two different extraction methods (direct transfer formic acid method on spot and ethanol formic acid extraction method) and different cut-offs for genus-/species-level identification were used. The novel strain was run in duplicate. Mass spectra were analysed in an m/z range of 2000 to 20 000. MALDI Biotyper library version 3.0 and MALDI Biotyper software version 3.1 were used for bacterial identification [5, 6].

The score cut-offs recommended by the manufacturer (≥2.000 for species-level, 1.700 to 1.999 for genus-level and <1.700 for no reliable identification) and lower cut-off scores (≥1.500 for genus-level, ≥1.700 for species-level and <1.500 for no reliable identification) were considered for identification. The MALDI-TOF analysis produced an unreliable performance score (<1700) and, therefore, failed to identify the micro-organism.

Analysis of the 16S rRNA gene was performed to confirm the phenotypic identification. A PCR product of the 16S rRNA gene, using the primers described by Weisburg *et al.* [7], was obtained with *Taq* DNA polymerase based on the manufacturer’s specifications (Promega). Sequencing of the 1.4 kb PCR products was performed on both DNA strands by the sequencing facility of Macrogen, Seoul, South Korea. The sequence was compared with known sequences using the blast v2.0 software (http://www.ncbi.nlm.nih.gov/BLAST/), which revealed 99 % sequence identity to the 16S rRNA gene (Genbank accession Y16426) of the type strain *Ignavigranum ruoffiae* CCUG 37658^T^. The Genbank accession number of the novel isolate is MG267060.

The susceptibility tests performed with antibiotic concentration gradient strips (Etest, bioMèrieux) on Mueller Hinton agar with sheep blood (5 %, v/v) showed the following values (µg ml^−1^): penicillin, 0.016; vancomicin, 0.25; ceftriaxone, 1; ciprofloxacin, 0.125; imipenem, 0.38; meropenem, 0.94; rifampicin, 0.004; cephalexin, 0.25 and TMS, 0.38. These values were compared with those in the reference methods described by the Clinical and Laboratory Standards Institute (CLSI) [broth dilution: CAMHB with LHB (2.5 to 5 %, v/v); agar dilution: MHA with sheep blood (5 %, v/v)] [8].

This case report here was presented at the XIX Lancefield International Symposium on Streptococci and Streptococcal Diseases, P0223, held in Buenos Aires, Argentina, in November 2014.

## Discussion

The arrangement of cells of our isolate in single cells, pairs and short chains excluded its classification in the genera *Pediococcus*, *Tetragenococcus*, *Aerococcus*, *Helcococcus*, *Dolosigranum* and *Alloiococcus*, whose cellular arrangement is in groups and tetrads*. Alloiococcus* morphology must be observed from agar plates because it does not grow in thioglycolate medium. *I. ruoffiae* is the only species of the genus *Ignavigranum* with similar morphology and related phenotypically to the genera *Leuconostoc*, *Weisella*, *Enterococcus*, *Vagococcus*, *Lactococcus*, *Streptococcus*, *Abiotrophia*, *Globicatella*, *Dolosicoccus*, *Gemella* and *Facklamia*, from which *Ignavigranum* must be differentiated. The genus *Ignavigranum* belongs to the family *Aerococcaceae*, order *Lactobacillales* of the class *Bacilli.*

The test results used to differentiate the genera *Facklamia* and *Ignavigranum* are sensitivity to vancomycin, no gas production from MRS glucose broth, negative test results for aesculin and bile-aesculin, and positive test results for PYR, LAP and growth with 6.5 % NaCl. *Dolosigranum pigrum* was excluded because aesculin hydrolysis is positive for this species [1, 4].

Isolates of *I. ruoffiae* and *Facklamia* have been studied in reference centres by different authors using the combination of molecular methods, conventional tests and miniaturized rapid tests. They have indeed found differences in the results of the identification [1, 3, 4, 9].

The tests that are useful in differentiating *I. ruoffiae* from *Facklamia* species are arginine deamination, hippurate hydrolysis and acid production from mannitol, sucrose, sorbitol and trehalose by conventional tests as shown in Table 1 adapted from the reports of Facklam [3] and LaClaire [4]. This combination of tests and the distinctive sauerkraut odour on blood agar allowed the identification of the isolate as *I. ruoffiae.*

*I. ruoffiae* is negative for hippurate hydrolysis, and the deamination of arginine is variable. Aesculin is not hydrolysed by any of the species except for some isolates of *Facklamia sourekii.*

The only two *Facklamia* species that are positive for arginine deamination are *Facklamia hominis* and *Facklamia miroungae;* however, both these species also hydrolyse hippurate whereas *I. ruoffiae* does not. In addition, the trehalose test is positive for *F. miroungae*, whereas *I. ruoffiae* tests negative. *F. miroungae* is represented by a non-human isolate recovered from a juvenile southern elephant seal [10].

*Facklamia languida*, which is hippurate hydrolysis and arginine deaminase negative, can be differentiated by acid production from trehalose [10]. *Facklamia tabacinasalis* can be differentiated from *I. ruoffiae* by acid production from sucrose and sorbitol [3]. *F. tabacinasalis* was isolated as a contaminant of powdered tobacco (snuff) [11].

This is the first case of a skin abscess caused by *I. ruoffiae* with a complete description of its microbiological and clinical features reported in Latin America.

It is necessary to emphasize the importance of the recovery and recognition of rare micro-organisms in mild and severe infections, and the importance of a polyphasic approach to arrive at the correct identification. In addition, the tests included in the rapid identification systems and conventional tests do not always correlate [4].

MALDI-TOF identification of *I. ruoffiae* was unreliable due to the fact that the taxon was not included in the database [6]. The phenotypic identification was confirmed with 16S rRNA gene sequence analysis.

Interpretative categories of antimicrobial susceptibility (resistant, intermediate, susceptible) were determined by using the CLSI guidelines for *Streptococcus* species other than *Streptococcus pneumoniae* [8] for penicillin and ceftriaxone. The interpretive standards for *S. pneumoniae* were used for presumptive susceptibility values for TMS, vancomycin, meropenem, imipenem and rifampicin. No interpretive standards are available for ciprofloxacin, cefalotin or cephalexin.

The novel strain seemed to be susceptible to the antimicrobials tested but had decreased susceptibility to carbapenems. It was fully susceptible to rifampicin compared with the susceptibility of different isolates of *Facklamia* species [12].

In summary, this case report provides more insights into the phenotypic characteristics and antimicrobial resistance profile of *I. ruoffiae.* In addition, it emphasizes the importance of the isolation, correct identification through a polyphasic approach and finally recognition of this infrequent and probable skin pathogen. It is important not to underestimate it or discard as contamination, or to consider this species as part of the resident microbiota of the skin.
